# Forearm fractures – are we counting them all? An attempt to identify and include the missing fractures treated in primary care

**DOI:** 10.1080/02813432.2023.2231028

**Published:** 2023-07-07

**Authors:** Cecilie Dahl, Eyvind Ohm, Siri Marie Solbakken, Nudrat Anwar, Kristin Holvik, Christian Madsen, Frede Frihagen, Åshild Bjørnerem, Frida Igland Nissen, Lene B. Solberg, Tone Kristin Omsland

**Affiliations:** a Department of Community Medicine and Global Health, University of Oslo, Institute of Health and Society, Oslo, Norway; b Department of Health and Inequality, Norwegian Institute of Public Health, Oslo, Norway; c Department of Physical Health and Ageing, Norwegian Institute of Public Health, Oslo, Norway; d Department of Orthopedic Surgery, Østfold Hospital Trust, Grålum, Norway; e Institute of Clinical Medicine, University of Oslo, Oslo, Norway; f Department of Clinical Medicine, UiT - The Arctic University of Norway, Tromsø, Norway; g Department of Obstetrics and Gynecology, University Hospital of North Norway, Tromsø, Norway; h Norwegian Research Center for Women’s Health, Oslo University Hospital, Oslo, Norway; i Department of Orthopedic Surgery, University Hospital of North Norway, Tromsø, Norway; j Division of Orthopedic Surgery, Oslo University Hospital, Oslo, Norway

**Keywords:** Forearm fractures, primary care, secondary care, incidence, Norway

## Abstract

**Objective:**

Norway has a high incidence of forearm fractures, however, the incidence rates based on secondary care registers can be underestimated, as some fractures are treated exclusively in primary care. We estimated the proportion of forearm fracture diagnoses registered exclusively in primary care and assessed the agreement between diagnosis for forearm fractures in primary and secondary care.

**Design:**

Quality assurance study combining nationwide data from 2008 to 2019 on forearm fractures registered in primary care (Norwegian Control and Payment of Health Reimbursement) and secondary care (the Norwegian Patient Registry).

**Setting and patients:**

Forearm fracture diagnoses in patients aged ≥20 treated in primary care (*n* = 83,357) were combined with injury diagnoses for in- and outpatients in secondary care (*n* = 3,294,336).

**Main outcome measures:**

Proportion of forearm fractures registered exclusively in primary care, and corresponding injury diagnoses for those registered in both primary and secondary care.

**Results:**

Of 189,105 forearm fracture registrations in primary and secondary care, 13,948 (7.4%) were registered exclusively in primary care. The proportion ranged from 4.9% to 13.5% on average between counties, but was higher in some municipalities (>30%). Of 66,747 primary care forearm fractures registered with a diagnosis in secondary care, 62% were incident forearm fractures, 28% follow-up controls, and 10% other fractures or non-fracture injuries.

**Conclusion:**

An overall small proportion of forearm fractures were registered only in primary care, but it was larger in some areas of Norway. Failing to include fractures exclusively treated in primary care could underestimate the incidence rates in these areas.

## Key points

Norwegian forearm fracture incidence based on secondary care may be underestimated by not including fractures treated exclusively in primary care.

The mean proportion of forearm fractures exclusively handled in primary care is 7% and varies from 5% to 14% between counties.

Fractures treated in primary care can be considered for more accurate national incidence rates. Correct fracture diagnosis needs further investigation.

## Introduction

Forearm fractures, including fractures of the distal forearm (wrist fractures) are the most common among fracture injuries [1,2], and also the most common type of fragility fracture [3]. After the age of 50 years, 50% of women and 20% of men will suffer a fragility fracture during their remaining lifetime [4]. Emergency care in Norway is organized as part of both primary and secondary health care [5], still estimations of incidence rates for fractures have only been based on figures from secondary care, such as local fracture registries [6], hospital osteoporosis centers[7] and medical records from hospital admissions or outpatient clinics [8]. The most recent age- and sex-adjusted estimate from national data on secondary care in persons ≥18 years old in Norway reported a distal radius fracture incidence of 244 per 100,000 [2]. This is comparable to the incidence in Sweden of 278/100,000 in persons ≥ 17 years old [9]. However, the incidence rate in Norway can be underestimated, as some fractures (in particular in young adults) are treated only in primary care [1,5].

The primary health care, run by the municipalities is responsible for general practitioners (GPs), and other care services, including the emergency care service. The emergency care service is the first line for most patients with injuries, and is organized as either municipal or inter-municipal (a collaboration between two or more municipalities), where most offer out-of-hour care [10]. Some primary emergency departments have access to x-ray services, for example if they are located near an alpine resort and have a long distance to nearest hospital [1]. Data are stored in the Norwegian Control and Payment of Health Reimbursement (KUHR) database. This database contains individual fracture diagnoses, suspected fractures that are not always confirmed with an x-ray, and follow-up controls that cannot easily be separated from acute events. Including all fracture registrations from KUHR may therefore overestimate the incidence.

The secondary care consists of hospitals, institutions and other services which, by referral, contribute in providing the correct diagnosis, e.g. using radiology. Patients with hip fractures are almost always admitted to a hospital for surgical treatment, and reliable incidence rates are therefore available based on figures from the Norwegian Patient Registry [11]. On the contrary, forearm fractures are often treated non-operatively in secondary or primary care, by an orthopedic surgeon or a GP at a municipal emergency care center. Stable, undisplaced, or minimally displaced fractures are usually treated with a cast, and treatment can often be carried out in a primary care facility. Due to the variation in treatment locations, forearm fractures are one of the most difficult fracture types to capture from registry-based data in Norway.

A previous forearm fracture almost doubles the risk of a subsequent fracture [12]. In Norway, many patients who have had their first fracture are not offered secondary fracture prevention [13]. Studies from other countries show that this care gap may be even larger in primary care, as osteoporosis assessment has been found to be suboptimal outside hospitals [14,15]. Identifying the proportion of forearm fractures that are treated exclusively in primary care and not included in the secondary care data would enable us to calculate more accurate national incidence rates, and it may also give an estimate of the extent of patients not being referred to hospital-based follow-up of fractures.

We aimed to (1) estimate the total number and proportion of forearm fractures registered exclusively in primary care in Norway and assess whether this proportion has varied over time and across sex, age and geography, and (2) assess whether primary care forearm fractures corresponded to fracture diagnoses in secondary care.

## Methods

### Pilot study

Little is known about registration practice in primary health care in Norway. Before undertaking this study, we therefore conducted a pilot study in five selected rural primary health care units located far from the nearest hospital and with x-ray machines available. In data from patients ≥20 years of age, 454 forearm fractures were obtained from the years 2015 and 2016, and 60.0% were incident fractures, 82% had an x-ray taken and 79.0% had their fracture treatment completed on site (see details in Appendix 1).

### Study population

We included all adult patients ≥ 20 years in Norway in the years 2008–2019, who were registered with a forearm fracture diagnosis in the primary care database KUHR, and with a relevant injury including fractures, sprains, contusions and dislocations in the secondary care database, the National Patient Registry (NPR). Adults 20–50 years were included to consider differences by age below and above 50 years.

### Primary care database

KUHR is based on claims from GPs and municipal emergency departments and uses the International Classification of Primary Care, Second edition (ICPC-2), which classifies a forearm fracture with the diagnosis code L72. This corresponds to the diagnosis code S52 in the 10th revision of the International Statistical Classification of Diseases and Related Health Problems (ICD-10) for forearm fracture, but does not discriminate on the site of fracture. Therefore, the ICD-10 code S52 with subgroups is sometimes used in primary care. KUHR also reports reimbursement codes for fracture treatment, which can be used as indication of an acute fracture event, however the codes are not always specific for a fracture, but can be used for sprains, strains and other injuries of the forearm (see Sensitivity analysis in Appendix 3).

### Secondary care database

NPR contains data from in- and outpatient records from all Norwegian hospitals and the emergency departments in some of the largest cities (Oslo, Bergen, Trondheim and Tromsø). The injury diagnoses were selected based on diagnosis codes overlapping with primary care forearm fractures in a previous study [5] (see Appendix 2). From NPR we included ICD-10 codes S52.0–S52.9 for forearm fracture, other fractures (e.g. S42, S62), follow-up (e.g. Z09.4) and other injury-codes (sprain, strain and dislocation (e.g.S53, S63) (Supplementary, Appendix 2), and NOMESCO Classification of Medical and Surgical Procedures [16].

### Statistics

**Figure 1.**
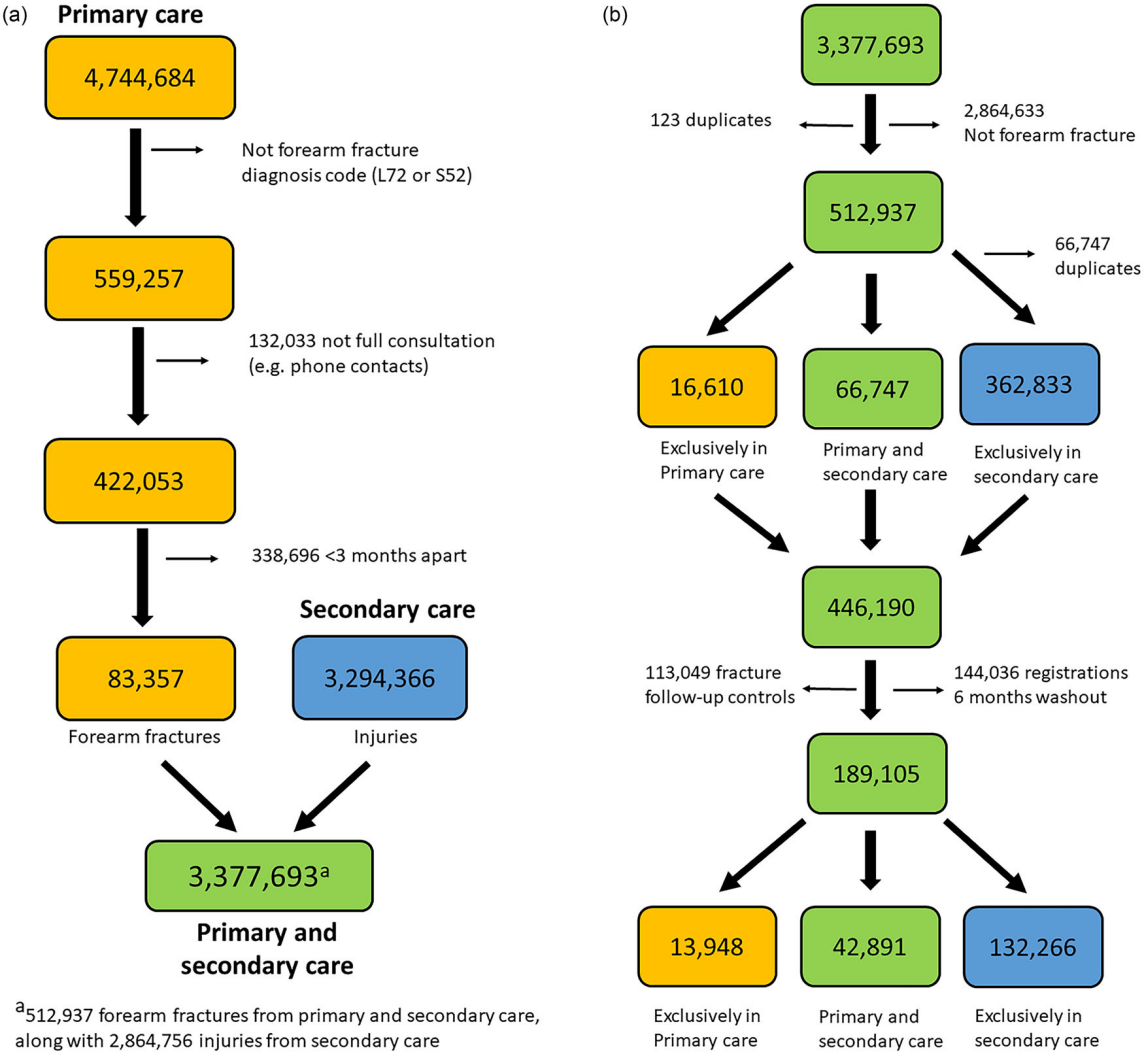
(a) Flow chart of the number of fracture records in primary care and injuries in secondary care (all diagnoses from Supplementary, Appendix 2). the orange boxes indicate registrations in primary care, the blue in secondary care, and the green in both databases combined. (b) Continuation of Figure 1(a), showing the number of forearm fracture records in the primary (orange), secondary (blue) and both databases (green) before and after 6 months washout.

Inclusion and exclusion when combining the databases: From primary and secondary care, forearm fracture diagnoses (ICPC-2 L72 and ICD-10 S52) were included, and diagnosis codes based on other contact types than ordinary medical consultations (e.g. phone consultations) and subsequent primary care forearm fracture registrations for the same individual within three months of the first registration were excluded (Figure 1(a)). We combined 83,357 primary care registrations of forearm fracture diagnoses with 3,294,336 secondary care registrations of forearm fracture diagnoses and other injury diagnoses. The databases were combined through the individual national identity number of each patient. The matching of secondary care diagnoses within a three-month period before or after a primary care forearm fracture registration were summarized in numbers and percent (*n*,%). The 3-months exclusion and matching window was chosen to reduce misclassification of type of diagnosis between primary and secondary care.

We included forearm fractures indicated in both the primary or secondary diagnoses in secondary care (subgroups S52.0–S52.9). In secondary care, an acute/incident forearm fracture was specified when there was no additional ICD-10 code for follow-up control or sequela (Z or T code, Supplementary, Appendix 2). Forearm fractures that had an additional registered medical or surgical procedural code, indicating fracture treatment (plastering, repositioning, and/or surgery) and those with no procedural code were included in the overall calculations.

Forearm fractures exclusively in primary care: The percentage of primary care forearm fractures with no corresponding diagnosis in secondary care was calculated as the number registered only in primary care divided by the total number of forearm fracture registrations in primary and secondary care (counting those in both registers only once, Figure 1(b)). In the final analysis, registrations with a follow-up control or sequela code (Supplementary, Appendix 2), and registrations occurring within 6 months in the same patients were excluded (‘washout’). Stata 16 was used for data cleaning, merging of files and analysis. A sensitivity analysis excluding fracture registrations without a reimbursement code for fracture treatment was also performed (Supplementary, Appendix 3).

## Ethics

The Norwegian Centre for Research Data (NSD, project number 587591) performed a Data Protection Impact Assessment, and the project (including the pilot study) was approved by the data protection officer at the University of Oslo as being pursuant to the General Data Protection Regulation. Exemption from consent for quality assurance was obtained from the Norwegian Directorate of Health (ref:19/3103-4). All data were stored on the research platform ‘Tjenester for Sensitive Data- TSD’, which meets all requirements of Norwegian law regarding safe handling and storage of sensitive data.

## Results

From primary care, there were 4,744,684 fracture diagnoses of any type from 2008-2019 (Figure 1(a)). After exclusion of other fracture types, phone consultations etc., and repeated fracture diagnoses within 3 months, 83,357 forearm fracture registrations remained (Figure 1(a)). From secondary care, there were 3,294,366 injury diagnoses registered, which were combined with the 83,357 forearm fractures from primary care (Figure 1(a)). After exclusion of other fracture types and duplicates also registered in primary care, a total of 446,190 forearm fracture diagnoses remained (16,610 only in primary care, 66,747 in both primary and secondary care and 362,833 only in secondary care, Figure 1(b)).

### Registrations exclusively in primary care

**Figure 2.**
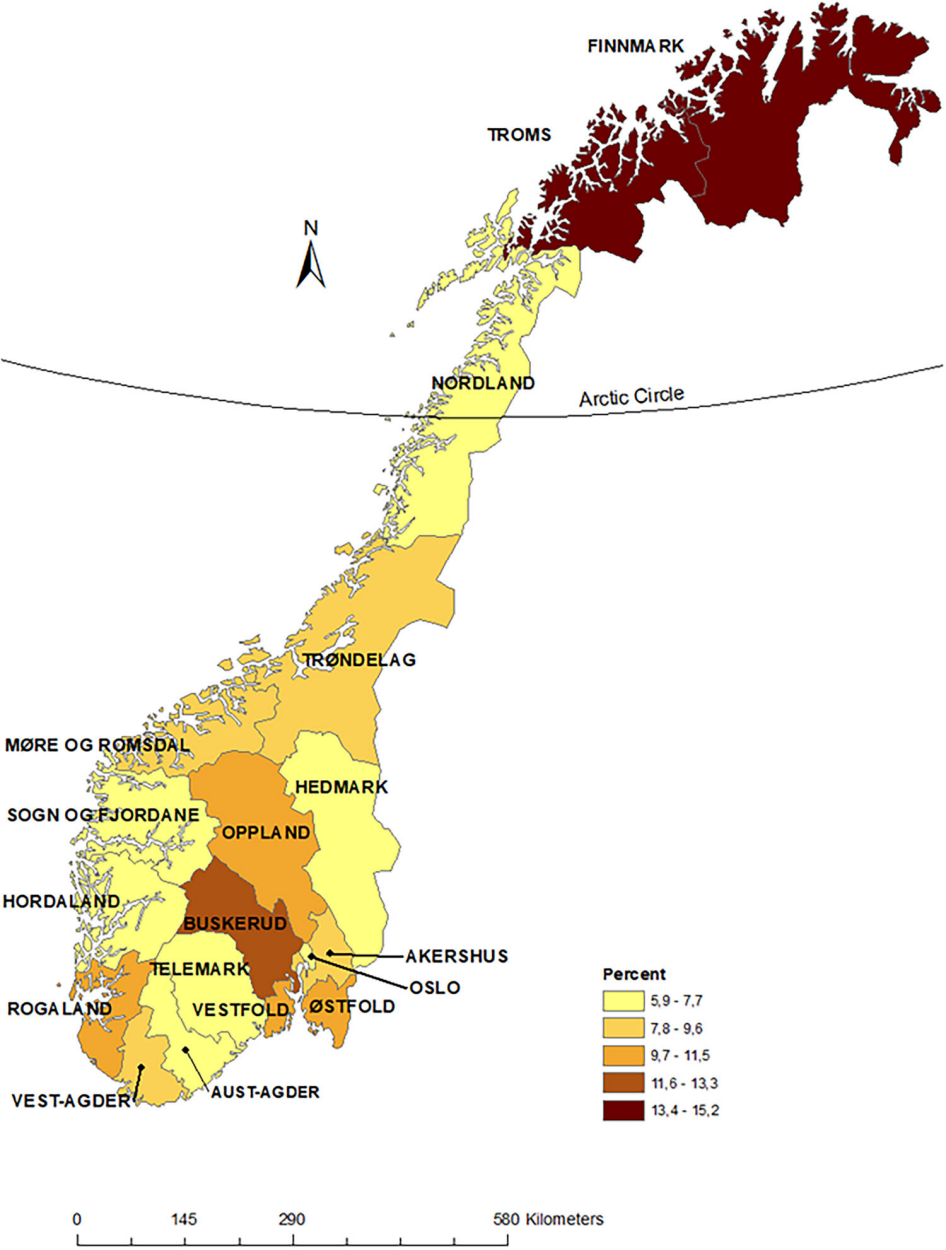
Percent forearm fractures registered only in primary care by 2019 County division of Norway.

**Figure 3.**
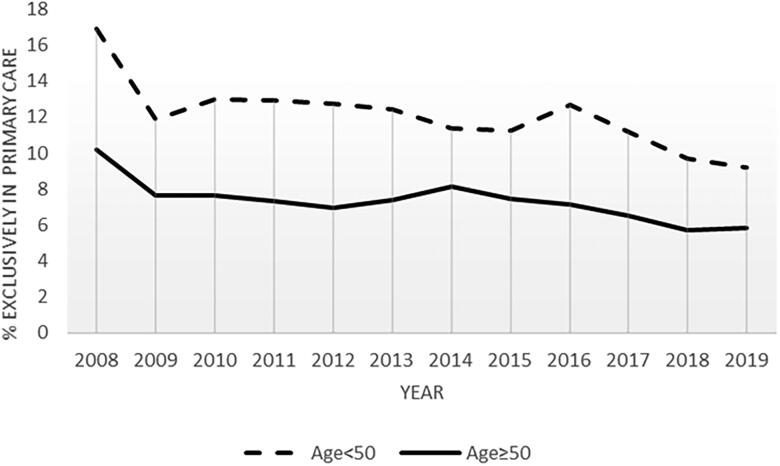
Percent of total forearm fractures registered only in primary care in the years 2008–2019. Stratified by age at fracture.

Of the total of 446,190 forearm fracture registrations, 16,610 (3.7%) (mainly L72) were found only in primary care (Figure 1(b) and Table 1). When excluding follow-up controls and forearm fracture registrations within the 6 months washout period in the combined dataset, 189,105 forearm fracture registrations remained (Figure 1(b)), and 13,948 (7.4%) were only in primary care. Men had a higher proportion (9.7%) treated exclusively in primary care compared to women (6.4%), and the youngest age group 20–49 years had a higher proportion (10.8%), compared to the oldest ≥50 years (6.0%) (Table 1). The highest proportion of forearm fracture diagnosis exclusively in primary care was in Troms and Finnmark counties 12.6% and 13.5% and the lowest in Hordaland with 4.9%, on average (Figure 2). At municipality level the range was from 0% to 30% (removing municipalities reporting <20 fractures per year, with few in secondary care). The mean annual number of forearm fractures treated only in primary care was 1162 fractures, and the number and proportion declined from 1657(11.0%) in 2008 to 930 (5.6%) in 2019 (Figure 3). If excluding the years 2008 and 2009, i.e. the start-up period of the secondary care register, the overall proportion was 7.0%.

**Table 1.** Number of forearm fracture registrations in the secondary care, and number and proportion registration only in primary care of a total of 446,190 forearm fractures registered from 2008-2019.

	Secondary Care^a^	Exclusively in primary care	
	n	n	%
All^b^ forearm fracture registrations	429,580	16,610	3.7
Acute^b,c^ forearm fracture registrations	316,531	16,610	5.0
All forearm fracture registrations, after washout^d^	183,526	13,948	7.1
Acute^b,c^ forearm fracture registrations, after washout^d^	175,157	13,948	7.4
Men^b,c,d^	50,163	5,413	9.7
Women^b,c,d^	124,994	8,535	6.4
Age < 50 years^b,c,d^	48,419	5,483	10.8
Age ≥ 50 years^b,c,d^	126,738	8,103	6.0
Health Trust North Norway^b,c,d^	15,797	1,554	9.0
Health Trust Mid Norway^b,c,d^	24,997	1,744	6.5
Health Trust West Norway^b,c,d^	34,567	2,366	6.4
Health Trust South-East Norway^b,c,d^	99,347	8,020	7.5
General practitioner^b,c,d^	8,606	11,555	57.3
Emergency units^b,c,d^	13,299	2,188	14.3
Hospitals (including large emergency departments)^b,c,d^	153,252	204	0.1

^a^Some registrations were in both the secondary and primary care databases, but were counted only once in secondary care.

^b^Primary and secondary diagnoses.

^c^Registrations with post-fracture follow-up code in secondary care excluded.

^d^6 months wash-out of total dataset (primary and secondary care).

### Matching registrations in primary and secondary care

A total of 66,747 (80.0%) of records in primary care matched with a secondary care diagnosis 3 months forward or backward in time. In 62.2% of the 66,747, the matching diagnosis in secondary care was a forearm fracture (S52), 35.3% had a medical procedural code for fracture treatment, whereas 5.2% did not have a procedural code (Table 2). A follow-up control or sequela diagnosis code was found in 28.2%, and an additional 7.6% had a follow-up control or sequela code along with a forearm fracture code. Other common diagnoses were: ‘fracture of the hand and wrist’ (S62), ‘contusion of the hand and wrist (S60)’, ‘fracture of the upper arm’ (S42), ‘contusion of the forearm’ (S50), and ‘sprain, strain and dislocation of hand or wrist’ (S63). In 78.0% of forearm fracture registration at both primary and secondary care, the primary care registration came before the secondary care, and the median was 6 days (interquartile range (IQR): 0-20). The median time from a secondary care registration to a primary care registration was 9 days (IQR: 1-28). There was on average three forearm fracture registrations (median, IQR: 1-5) per patient, including both the primary and secondary care registrations, with a maximum of 325 registrations before washout.

**Table 2.** ICD-10 diagnoses in secondary care that are matching 66,747 forearm fracture diagnosis registered in primary care.

	Total matching		Primary care first		Secondary care first	
ICD-10 diagnosis code as primary or secondary diagnoses	*n*	%	*n*	%	*n*	%
Any match with relevant codes^a^	66,747	100.0	51,953	77.8	14,794	22.2
Forearm fracture (S52)	41,498	62.2	3,3190	49.7	8,308	12.4
Acute forearm fracture with procedure code for treatment^b^	23,592	35.3	20,619	30.9	2,973	4.5
Acute forearm fracture, without procedure code for treatment^c^	3,439	5.2	2,165	3.2	1,274	1.9
Follow-up control or sequela^d^	18,828	28.2	12,391	18.6	6,437	9.6
Follow-up control or sequela^d^ with S52 code as primary or secondary diagnosis	5,092	7.6	2,661	4.0	2,431	3.6
Follow-up control or sequela^d^ *without* S52 code as primary or secondary diagnosis	13,736	20.6	9,730	14.6	4,006	6.0
**ICD-10 diagnosis code as primary diagnoses:**						
Any match with relevant codes^a^	66,747	100.0	51,953	77.8	14,794	22.2
Forearm fracture (S52)	37,820	56.7	31,123	46.6	6,697	10.0
Fracture of the hand or wrist (S62)	1,743	2.6	1,437	2.2	306	0.5
Fracture of the upper arm (S42)	1,408	2.1	1,160	1.74	248	0.4
Sprain, strain and dislocation of forearm (S53)	514	0.8	415	0.6	99	0.1
Sprain, strain and dislocation of hand or wrist (S63)	800	1.2	577	0.9	223	0.3
Sprain, strain and dislocation of upper arm and shoulder (S43)	200	0.3	160	0.2	40	0.1
Contusion of the forearm (S50)	1,171	1.8	773	1.2	398	0.6
Contusion of the hand or wrist (S60)	1,502	2.3	1,075	1.6	427	0.6
Contusion of the upper arm (S40)	222	0.3	172	0.3	50	0.1
Osteoporosis after menopause, with pathological fracture (M80)	630	0.9	434	0.7	196	0.3
Osteoporosis without pathological fracture (M81)	285	0.4	222	0.33	63	0.1
Disruption of bone continuity, incorrect healing (M84)	232	0.3	165	0.3	67	0.1
Other disorders of bone (M89)	139	0.2	111	0.2	28	0.0
Other primary diagnosis	3,456	5.2	2,803	4.2	653	1.0
Unknown primary diagnosis	142	0.2	132	0.2	10	0.0

^a^See Appendix 2 for relevant codes (e.g. S62, S42, S60, S40, S50).

^b^No follow-up control code, but registered procedure code indicating plastering, repositioning or acute prosthesis.

^c^Only one fracture registration in NPR and no follow-up control code, but without procedure code indicating fracture treatment registered.

^d^ICD-10 diagnoses (with subgroups*) indicating follow-up or sequela:.

T81* Bleeding and hematoma as complication to surgical or medical procedure.

T84* complication to surgical or medical procedure.

T88.8 Other specified complications to surgical or medical procedure, not specified elsewhere.

T88.9 Other unspecified complications to surgical or medical procedure, not specified elsewhere.

T92*Sequelae after injury to the upper extremity.

Z04.8 Examination and observation for another specified cause.

Z09.4Follow-up control after fracture.

Z09.0 Follow-up control after surgical treatment for other reasons.

Z09.7 Follow-up control after combination treatment for other conditions.

Z09.8 Follow-up control after other specified treatment for other conditions.

Z09.9 Follow-up control after other unspecified treatment for other conditions.

Z44.8 Adaptation and adjustment of another specified external prosthesis.

Z44.9 Adaptation and adjustment of another unspecified external prosthesis.

Z45.8 Adjustment and control of other specified implanted equipment.

Z45.9Adjustment and control of other unspecified implanted equipment.

Z46.7 Adaptation and adjustment of orthopedic aids.

Z46.8 Adaptation and adjustment of other specified aids.

Z46.9Adaptation and adjustment of other unspecified aids.

Z47* Contact with health care services for other orthopedic follow-up.

Z48* Contact with health care services for other follow-up after surgery.

Z50* Contact with health care services for rehabilitation treatment.

Z54* Contact with health care services for recovery purposes.

ZXD 10 (NCSP- code) Elective intervention.

## Discussion

In this study, most patients with a forearm fracture diagnosis in primary care also had a forearm fracture diagnosis in secondary care. However, 7.4% of the registrations were found exclusively in primary care. The percentage of registrations exclusively in primary care was highest in men, the youngest age group (<50 years), in the North of Norway, and it declined over time.

This is the first time that the number and percentage of forearm fractures treated only in primary care has been estimated in Norway. We studied whether fracture diagnoses in primary care (often based on suspicion) corresponded with fracture diagnoses in secondary care. We had access to a large range of injury diagnoses and diagnosis codes at follow-up, making it unlikely that the estimated percentage treated only in primary care was due to missing diagnoses in our secondary care data.

There are some limitations. The secondary care data are not perfectly complete or correct, and studies of the sensitivity and positive predictive value (PPV) of forearm fracture diagnosis in secondary care according to the x-ray gold standard are ongoing. The compliance between ICPC diagnoses codes and medical records have been found to be around 85% for consultations in primary care in Norway, and may be higher for fractures if they are assessed by x-ray on-site [17]. Still, a fracture diagnosis code is often set on suspicion of fracture in primary care. Therefore, we do not know what proportion of fracture diagnoses were tentative or follow-up controls, which means that the exact number of acute fractures treated only in primary care was unfortunately not possible to determine in the current study.

ICPC-2 reimbursement codes should be reported in primary care for refund of treatment. In the pilot study, however, we found that the registration of reimbursement codes was inconsistent (Supplementary, Appendix 1). Moreover, the reimbursement codes themselves are not specific for fracture treatment (i.e. a sprain may receive the same treatment and code as a suspected fracture, Supplementary, Appendix 3). Reliable and specific medical procedure codes in primary care (KUHR) would have improved our assessment, but these are not mandatory to report, and therefore not reported on a regular basis. Further validation studies of primary care data are warranted.

The proportion of fractures treated only in primary care (7.4%) was lower compared to a previous Norwegian study on injuries treated in primary and secondary care [5], where 31.0% of all fractures were treated exclusively in primary care; however, this percentage was only 14.4% for forearm fractures (personal communication with E. Ohm and not reported in that study [5]). The discrepancy may be due to the different methods as they did not include secondary diagnoses of fractures from secondary care and they counted only one injury episode per calendar year [5]. This is likely to reduce the match, and thereby increase the proportion exclusively in primary care. Moreover, they also included pediatric fractures, where a higher proportion of the youngest children (0–9 years) had been treated for their injuries exclusively in primary care [5]. In the current study, we included *all* registered primary (principal) and secondary diagnosis in adults (≥20 years) only, and we used a continuous moving matching window of 6 months (i.e. 3 months forward and backward in time) rather than one per calendar year.

In Norway, primary care and large emergency units in the urban areas (included under secondary care) have the main responsibility for fracture traumas [18]. Admissions to hospital care and radiology are principally by referral, and if the health care provider has an x-ray unit available, the threshold for referring patients from rural primary care units to hospital is high [1]. Our pilot study showed that several acute fractures were treated on site after detection with x-ray and without referral to hospital, which means that a reimbursement for fracture was indicated. Still these codes were not consistently reported by the GPs. This is an important implication for future use of the primary care data, as inclusion of fracture registrations with reimbursement codes only will most likely underestimate the number from primary care.

The high number of registrations for each fracture event necessitates the application of a washout period. Not to be confused with the general washout of the entire dataset, we initially applied a clinically recommended exclusion of repeated registrations within three months, to be able to investigate secondary care diagnoses registered three months forward and backward in time of a primary care diagnosis. Still, it should be kept in mind that application of a short washout period of primary care records will most likely lead to an inclusion of several non-fracture injuries. In the current study, 18% of primary care registrations were less likely to represent a fracture as the primary diagnosis indicated non-S52-conditions in secondary care. To obtain a ‘worst case scenario’ of the proportion treated exclusively in primary care, we chose an overall washout of 6 months for the total dataset. This was based on numbers from a study of record-verified forearm fractures, where two acute forearm fractures occurred within 6 months in approximately 2.0% of all patients [19]. Given the same risk of subsequent forearm fracture in the current study, the proportion of tentative or follow-up diagnoses are likely to be substantially larger than the proportion with two acute forearm fractures within this time window. Consequently, the true proportion treated only in primary care is likely to be closer to the 6-month washout estimate, because this data set will retain most incident fractures while excluding most follow-up controls

A higher proportion of exclusively primary care forearm fractures were found in the North and in rural areas of Norway. In areas with far distance to hospital and high reliance on primary care, it is possible that more fractures will be treated non-operatively (‘conservative’). There is currently no consensus on which treatment method is best, except for unstable, displaced fractures where operative treatment is indicated [20,21]. We have limited knowledge about the treatment of forearm fractures in primary care in Norway, however several general practitioners report that they often consult an orthopedic surgeon before deciding on the path of treatment (personal communication with GPs during the pilot study and [1]). In a study of U.S. Medicare patients, conservative treatment varied by geography, and interestingly, this was independent of the density of orthopedists in the region [22]. A higher proportion of exclusively primary care forearm fractures may therefore not necessarily mean poorer quality of treatment. Still, treatment exclusively in primary care could influence the extent of follow-up after a fracture. As in other countries, there is a large treatment gap of osteoporosis in Norway [13,23]. Previous studies suggest that treatment of forearm fractures exclusively in primary care rarely leads to an evaluation for osteoporosis [14,15], however we do not know whether this also applies to Norway.

### Future recommendations

To our knowledge, no national fracture registers include fractures treated exclusively in primary care. In Norway, a prospective nationwide fracture register also including fractures treated solely in primary care would be resource-demanding, and still probably have a risk of low completeness, due to challenges with many small primary care facilities treating a low number of fractures each. However, changes in registration procedure, such as digitalization of x-rays (teleradiology), digital communication and secondary assessments by radiologists and orthopedic surgeons, has many places become the present standard of non-operative fracture handling, which could increase registration in secondary care. In 2017, a new linkage between KUHR and other primary care registries were made with the aim to improve data quality, i.e. the Norwegian Registry for Primary Health Care (NRPC or KPR in Norwegian [24]. This may also improve future registration.

From 2008 to 2019 there were over 4.7 mill primary care fracture registrations that the current results may be applied to. Due to the high amount of tentative fracture diagnoses in the primary care registry that are not possible to separate from acute events, our recommendation for future studies is that using only secondary care data may still be the best option. Potential selection bias, in particular if studying associations by geography and over time, should be kept in mind, and if studying incidences (i.e. absolute rates), weighting techniques can be applied to account for the proportion lost. Different quality assurance processes can also be considered, for example would the inclusion of all first-registrations within 6 months (even if a follow-up code was given in secondary care) capture a large portion of fractures first presenting in primary care. If choosing to use the primary care register as an additional source to the secondary data (e.g. when studying incidences), only medical consultations (not simple contacts, such as phone consultations) should be included, and a washout period should be applied. The registrations should not be restricted to those with reimbursement or procedural codes for treatment, as this would underestimate the incidences.

## Conclusion

In this study of primary and secondary care registered forearm fractures, an overall 7.4% were registered with a forearm fracture diagnoses exclusively in primary care. Some of these may be tentative fracture diagnoses or non-fracture injures that are not possible to separate from acute events. The proportion of acute fractures treated only in primary care is overall small and may be overestimated, however it was larger in some Norwegian regions than others. Most forearm fractures presenting in primary care also received a forearm fracture diagnosis in secondary care (60.0%), however 35% received a follow-up control code. Future studies with more accurate diagnosis of forearm fracture based on x-ray records obtained in both primary and secondary care are warranted to optimize the calculation of national incidence rates.

## Supplementary Material

Supplemental Material
