# Supplementary material for: Forearm fractures – are we counting them all? An attempt to identify and include the missing fractures treated in primary care
Source: Scand J Prim Health Care. 2023 Jul 7;41(3):247–56. doi: 10.1080/02813432.2023.2231028 (PMC10478616; doi:10.1080/02813432.2023.2231028)
Supplement: Supplemental Material [file IPRI_A_2231028_SM6214.docx]

## **Supplementary material**

## Appendix 1. Pilot study

**Methods**

Before conducting the study, we contacted five rural health care units: Trysil and Hallingdal/Ål health units in the mountainous South-East, and Nordreisa, Lenvik and Alta health units in the North. These units were located far from the nearest hospital (1-3.5 hours’ drive) and therefore presumably had a higher threshold for referring patients to secondary care, compared to units in urban areas. All these health care units had radiological services available. A medical student (NA) travelled to the sites (except for Trysil and Alta, who sent anonymous data), and collected data from patients aged ≥20 years, who presented with forearm fractures in 2015 and 2016. The diagnoses codes (ICPC-2 L72 and ICD-10 S52), age group (five-year age intervals), sex, whether the consultation was for a new/acute fracture or a control, if an x-ray was taken on site, and other relevant information on treatment and referrals was obtained.

**Results**

A total of 453 forearm fracture registrations were collected from the rural health care units (Table S1). Most patients (64.3%) were ≥50 years of age, and 72.0% were women. There were 271 (60.0%) acute fracture registrations, where 221 (82.0%) of the acute fracture patients had an x-ray taken on site, and 214 (79.0%) had their fracture treatment completed on site, according to their medical records. Hence, 214 of 454 fractures (47.0%) would subsequently not occur in the secondary care registry. Of 214 reported acute fractures with x-ray taken and treatment being completed on site, 104 (78.0%) had a reimbursement code 106a or 106b. For those with non-acute fractures (follow-up controls and non-fracture injuries), 14 (9.2%) had the code 106a (plastering and dressing).

## Table S1. Characteristics of 454 fracture registrations obtained in pilot study at five rural health care units

| Information from medical records |  | Number | Percent |
| --- | --- | --- | --- |
| Gender | Male | 127 | 28.0 |
|  | Female | 326 | 72.0 |
| Age group | 20-50 | 162 | 35.7 |
|  | 50-60 | 99 | 21.8 |
|  | 60-70 | 99 | 21.8 |
|  | 70-80 | 55 | 12.1 |
|  | 80-103 | 38 | 8.4 |
| Health care unit | Alta | 110 | 24.3 |
|  | Finnsnes | 64 | 14.1 |
|  | Nordreisa (Sonjatun) | 91 | 20.0 |
|  | Hallingdal/Ål | 105 | 23.1 |
|  | Trysil (only year 2016) | 84 | 18.5 |
| Acute fracture | Yes | 271 | 59.8 |
|  | No | 153 | 33.8 |
|  | missing | 29 | 6.4 |
| Fracture follow up control | Yes | 147 | 32.5 |
|  | No | 216 | 47.7 |
|  | missing^a^ | 90 | 19.9 |
| Referred to hospital | Yes | 63 | 13.9 |
|  | No | 360 | 79.4 |
|  | missing | 30 | 6.2 |
| Fracture treatment completed on site | Yes | 324 | 71.5 |
|  | No | 101 | 22.3 |
|  | missing | 28 | 6.4 |
| X-ray taken on site | Yes | 288 | 63.6 |
|  | No | 136 | 30.0 |
|  | Missing | 29 | 6.6 |
| Billing code | 106a | 104 | 22.9 |
|  | 106b | 51 | 11.2 |
|  | 106a and 106b | 10 | 2.2 |
|  | missing | 288 | 63.6 |
| ^a^ Trysil site misinterpreted “follow-up control” (i.e. recorded those that were referred to follow up rather than those that came for follow-up. Data from Trysil were therefore set as missing) | | | |

## Appendix 2: Diagnoses codes obtained from specialist care

### 1. ICD-10 Diagnoses (injury)

### * including all subgroups

S22 * Fracture of the thoracic spine and thorax

S32 * Fracture of the lumbar spine and pelvis

S40 * Contour upper arm

S42 * Fractures of the humerus, scapula and clavicula

S43 * Dislocation, sprain and strain of joints and ligaments in the shoulder arch

S50 * Contour forearm

S52 * Forearm fracture

S53 * Dislocation, sprain and strain of joints and ligaments in the elbow

S60 * Contusion hand

S62 * Hand and finger fracture

S63 * Wrist dislocation

S72 * Fracture of femur

S82 * Leg fracture

S92 * Fracture of feet and toes

T02 * Fracture involving several body regions

T08 * Columna vertebralis, unspecified part

T10 * Upper extremity fracture, unspecified part

T12 * Fracture of lower extremity, unspecified part

M48.4 Fatigue fracture in vertebra

M48.5 Collapsed vertebrae, not elsewhere classified

M80 * Osteoporosis with pathological fracture

M81 * Osteoporosis without pathological fracture

M82 * Osteoporosis with underlying disease

M83 * Osteomalacia

M84 * Improper fracture healing

M85 * Other disorders of bone density and bone structure

M86 * Osteomyelitis

M89 * Other disorders of bone

### 2. ICD-10 Diagnoses (complications and codes used to determine whether fractures are new/incident)

* including all subgroups

T81 * Bleeding and hematoma as a complication of surgical and medical procedures

T84 * Complications of surgical and medical procedures

T88.8 Other specified complications for surgical and medical treatment, not elsewhere

specified or included

T88.9 Other unspecified complications of surgical and medical treatment, not elsewhere

specified or included

T92 * Sequelae of upper extremity injuries

T93 * Sequelae of lower extremity injuries

Z04.8 Examination and observation of other specified cause

Z09.0 Follow-up examination after surgical treatment for other conditions

Z09.7 Follow-up examination after combination therapy for other conditions

Z09.8 Follow-up examination after other specified treatment for other conditions

Z09.4 Follow-up examination after fracture

Z76.9 Contact the health service under other unspecified circumstances

Z09.9 Follow-up examination after other unspecified treatment for other conditions

Z44.8 Adaptation and adjustment of other specified external prosthesis

Z44.9 Adaptation and adjustment of unspecified external prosthesis

Z45.8 Adjustment and control of other specified implanted device

Z45.9 Adjustment and control of unspecified implanted device

Z46.7 Adaptation and adjustment of orthopedic aids

Z46.8 Adaptation and adjustment of other specified aids

Z46.9 Adaptation and adjustment of other unspecified aids

Z47 * Contact the health service for other orthopedic finishing

Z48 * Contact the health service for other post-surgery post-treatment

Z50 * Contact the health service for treatment that includes rehabilitation measures

Z51.8 Other specified treatment measures

Z51.9 Other unspecified treatment measures

Z54 * Contact the health service for recovery purposes

Z76.8 Contact the health service under other specified circumstances

Z76.9 Contact the health service under other unspecified circumstances

## Appendix 3. Sensitivity analysis: Exclusion of fractures without reimbursement codes

**Background**

The current sensitivity analysis was based on the definition of the Norwegian reimbursement codes used in 2015:

106a: plaster and dressing, i.e.:

- Zinklim dressing («Zinklimbandasje»)
- Plastering of a fractura claviculae. Repositioning of a dislocated jaw
- Immobilization of a fracture without dislocation, e.g. fissura radii/ tibiae/ antebrachii in children
- Temporary plastering of dislocations and fractures in large joints and bones
- Repositioning of a dislocated shoulder or elbow
- Change of dressing
- Change of plaster-dressing on the forearm and hand, and at the extremities in children

106b: Treatment of fractures that require repositioning under anesthesia and x-ray control before and after, for example distal forearm fracture.

The complete version of the reimbursement codes for the year 2014-2015 can be found here: <http://www.dokter.no/PDF-filer/Fastlegetariff_2014.pdf> (page 25)

The current reimbursement codes (2022-2023) can be found here: <https://normaltariffen.legeforeningen.no/>

Procedural codes and follow-up codes are not consistently registered in the primary care database, but reimbursement codes may indicate treatment of an acute fracture . Reporting of reimbursement codes will increase refund for treatment; however, it is uncertain whether reimbursement codes are consistently being reported.

**Methods**

We performed a sensitivity analysis excluding forearm fracture registrations (L72) in primary care that did not have a registered reimbursement code (i.e. 75% of registrations), comparing the remaining registrations to those exclusively registered in primary care in the main analysis.

**Results**

When excluding diagnoses without reimbursement codes in primary care, there were 1,301 forearm fracture registrations exclusively in primary care, which constituted 0.7% of total (primary and secondary care) registrations.

The pattern across sex (i.e. higher proportion of exclusively primary care registered fractures in men), age (i.e. higher proportion in the youngest), geography (i.e. higher proportion in the North) and calendar time (i.e. lower proportion in recent years) was similar to the main analysis. Most primary care diagnoses (78.0%) overlapped with an acute forearm fracture diagnosis in secondary care (main analysis: 62.0%), whereas 17.0% overlapped with a follow-up control code (main analysis: 28.0%).

**Conclusion**

Inclusion of only fractures with reimbursement code in primary care will result in a higher proportion of acute fractures relative to follow-up controls. However; as only 25% of fractures had a reimbursement code registered, the proportion of acute fractures included overall will most likely be too low compared to the actual number of acute fractures treated in primary health care.
